# Altered Brain Activity in Strabismic Amblyopic Children as Determined by Regional Homogeneity: A Resting-State Functional Magnetic Resonance Imaging Study

**DOI:** 10.3389/fnins.2022.879253

**Published:** 2022-06-02

**Authors:** Si-Wen Tan, Guo-Qian Cai, Qiu-Yu Li, Yu Guo, Yi-Cong Pan, Li-Juan Zhang, Qian-Min Ge, Hui-Ye Shu, Xian-Jun Zeng, Yi Shao

**Affiliations:** ^1^Department of Ophthalmology, The First Affiliated Hospital of Nanchang University, Nanchang, China; ^2^The First Clinical Medical College, Nanchang University, Nanchang, China; ^3^Department of Radiology, The First Affiliated Hospital of Nanchang University, Nanchang, China

**Keywords:** strabismus amblyopia, regional homogeneity, resting-state functional MRI, children, ReHo

## Abstract

**Objective:**

Earlier research has determined that amblyopia or strabismus may cause remarkable brain anatomical and functional variations. Nonetheless, thus far, the spontaneous changes in brain activity in children with strabismus amblyopia (SA) remain unclear. The purpose of this study was to determine the association between abnormal brain activity in children with SA and its behavioral manifestations.

**Patients and Methods:**

?A total of 24 children with SA (10 male and 14 female children) as well as 24 healthy controls (HCs), including 10 male and 14 female children were closely matched in sex and age, and examined using resting-state functional magnetic resonance imaging (fMRI). The regional homogeneity (ReHo) technique was applied to evaluate spontaneous cerebral activity variations in children with SA and HCs. Moreover, associations between altered ReHo values in distinct cerebral areas and the degree of strabismus were assessed using Pearson correlation analysis.

**Results:**

Remarkably increased ReHo values were observed in the right lingual, right superior frontal medial, bilateral superior parietal, and right inferior parietal gyri of children with SA compared with HCs. In contrast, mean ReHo values in children with SA were lower in the right cerebellum, left superior frontal gyrus, and left putamen nucleus. Furthermore, esotropia showed a positive correlation with ReHo values of the left putamen.

**Conclusion:**

The anomalous spontaneous activity changes in several brain areas that are caused by SA may indicate neuropathologic mechanisms of visual deficits and oculomotor disorders in children with SA.

## Introduction

**FIGURE 1:**
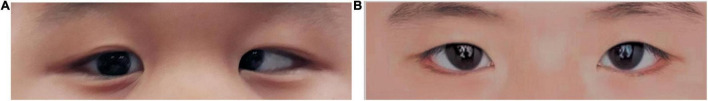
Eyes of children with strabismus amblyopia **(A)** and healthy children **(B)**.

Strabismus and amblyopia are two common ocular diseases. Strabismus is an ophthalmic disease owing to the disorder of extraocular muscles, which is considered relevant to the dysplasia of cerebral visual pathways that regulate eye movements. Both the eyes of the strabismus patients cannot focus on the target at the same time, and the optical axes of both eyes are separated. In addition, amblyopia is a visual disorder caused by ocular maldevelopment, which can be detected by decreased visual acuity and sensitivity. Still, there were no organic lesions in the eye examination. Strabismus amblyopia (SA) is one form of amblyopia caused by strabismus. During the early phase of visual development, strabismus can cause the production of two separate images by the eyes that do not coincide, leading to abnormal vision, including double vision or visual confusion (Figure 1). In such cases, nerve impulses relayed by squinting would be suppressed by the brain. Over time, long-term suppression would lead to the development of amblyopia (Korah et al., 2014).

In pediatric populations, strabismus reportedly has a marked influence on the development of amblyopia. The combined condition, SA, leads to functional deficiency, including defective motor, visual, and sensory cognition as well as impaired stereoscopic depth perception (Levi et al., 2015). These inadequacies are evidenced by imprecision or incompetence in reading, grasping, or driving, which will affect the quality of the patient’s daily life.

Functional magnetic resonance imaging (fMRI) is a common method that can precisely detect brain function. Its main advantage compared with traditional MRI is its ability to display subtle microscopic structural differences and satisfactory spatial resolution. When a person looks at an object, light passes through the retina, and nerve impulses are relayed along the visual pathway to the cerebral cortex, which produces the corresponding cortical activity to generate vision. This cortical activity can be observed and recorded by fMRI. Patients with SA differ from persons with normal vision in terms of the location, range, and degree of activation in cortical areas, which can also be detected by fMRI technology. Previously, fMRI techniques have been applied to detect cerebral activity alternations in either amblyopia or strabismus patients, separately (Lee et al., 2001; Chen and Tarczy-Hornoch, 2011).

The regional homogeneity (ReHo) is an extensively applied method that belongs to the resting-state fMRI (rs-fMRI), which is deemed dependable and accurate. Previous studies have shown that it has a high neurobiological relevance and test-retest reliability. A decline in ReHo values represents reduced synchrony and disordered activity. However, an increased ReHo value suggests increased synchrony of spontaneous neuronal activity. The ReHo technique has been successfully used in many researches on eye disorders (Cui et al., 2014; Song et al., 2014, 2017; Shao et al., 2015; Guo et al., 2016; Huang et al., 2016, 2017; Tang et al., 2018; Shi et al., 2019; Xu et al., 2019; Zhang et al., 2020; Tong et al., 2021; Yu et al., 2021; Table 1), along with many neurogenic diseases, like Parkinson’s disease (Dai et al., 2012) and sleep disorders (Li et al., 2016).

**TABLE 1:** ReHo method applied in ophthalmological diseases (partially).

Author	Year	Disease	Brain areas	
			
			UDS > HCs	UDS < HCs
Song et al., 2014	2014	Glaucoma	RDACC, MFG, RCAL	Calcarine, PG, LIPL, LCPL
Cui et al., 2014	2014	Diabetic retinopathy	PLC, ACC, FL	OL, PG
Shao et al., 2015	2015	Optic neuritis	LFG, RIPL	LCPL, LMTG, RI, RSTG, LMFG, ACC, MFG, SFG, RPG
Huang et al., 2016	2016	Comitant strabismus	RITC/FG/CAL, RLG, CG	
Song et al., 2017	2017	Pituitary adenoma	LSOG, MOG	LIFG, RMTG
Huang et al., 2017	2017	Retinal detachment		ROL, RSTG, cuneus, LMFG
Tang et al., 2018	2018	Acute eye pain	LSFG, RIPL, LP	PG, LMFG
Shi et al., 2019	2019	Exotropia	V2	BA47
Xu et al., 2019	2019	Corneal ulcer	CPL, LITG, RLG, LMFG, LAG, LCG, RAG, SFG	RAC, LPG
Zhang et al., 2020	2020	Diabetic vitreous hemorrhage	CPL, RS/MOG, SFG	RI, MFG
Tong et al., 2021	2021	Iridocyclitis		RIOG, calcarine, RMTG, RPG, LSOG, LP
Guo et al., 2016	2021	Diabetic optic neuropathy	RMFG, LAC, SFG/LFSO	
Yu et al., 2021	2021	Dry eye	MFG, IFG, SFG	

*HCs, healthy controls; RDACC, right dorsal anterior cingulated cortex; MFG, medial frontal gyrus; RCAL, right cerebellar anterior lobe; PG, precuneus gyrus; LIPL, left inferior parietal lobule; LCPL, left cerebellum posterior lobe; PLC, posterior lobe of cerebellum; ACC, anterior cingulate cortex; FL, frontal lobe; OL, occipital lobe; LFG, left fusiform gyrus; RIPL, right inferior parietal lobule; LMTG, left middle temporal gyrus; RI, right insula; RSTG, right superior temporal gyrus; LMFG, left middle frontal gyrus; SFG, superior frontal gyrus; RPG, right precuneus gyrus; RITC/FG/CAL, right inferior temporal cortex/fusiform gyrus/cerebellum anterior lobe; RLG, right lingual gyrus; CG, cingulate gyrus; LSOG, left superior occipital gyrus; MOG, middle occipital gyrus; LIFG, left inferior frontal gyrus; RMTG, right middle temporal gyrus; ROL, right occipital lobe; LSFG, left superior frontal gyrus; RIPL, right inferior parietal lobule; LP, left precuneus; V2, the right secondary visual cortex; BA47, Brodmann area 47; CPL, cerebellum posterior lobe; LITG, left inferior temporal gyrus; LAG, left angular gyrus; LCG, left cingulate gyrus; RAG, right angular gyrus; RAC, right anterior cingulate; LPG, left precentral gyrus; CPL, cerebellar posterior lobes; RS, right superior; RI, right insula; RIOG, right inferior occipital gyrus; LP, left precuneus; RMFG, right middle frontal gyrus; LAC, left anterior cingulate; LFSO, left frontal superior orbital gyrus; IFG, inferior frontal gyrus.*

Here, the ReHo technique was applied to analyze the alternations of spontaneous cerebral activity between children with SA and healthy controls (HCs) and to determine the relevance between the altered ReHo values and abnormal vision.

## Patients and Methods

### Patients

Twenty-four children with SA, including 10 male patients and 14 female patients, from the Ophthalmology Department of the First Affiliated Hospital of Nanchang University, were recruited to participate in this study. The following are the inclusion criteria: (i) children under 12 years old; (ii) diagnosed with SA; (iii) with a best-corrected visual acuity (VA) ≥0.20 logMAR units, and central fixation of both eyes with greater than one line difference; and (iv) no other eye diseases (such as optic neuritis, cataract, or glaucoma, etc.). Patients meeting the following criteria were excluded: (i) had eye operation record (intraocular and extraocular were both included); (ii) had other disorders besides eye disease (such as ischemic disease, inflammation, or infection); (iii) had a mental disease or cerebral infarction; (iv) was either addicted to illicit drugs or was an alcoholic.

Twenty-four HCs matched to those basic clinical characteristics of the SA group, like sex and age were also incorporated in this research, including 10 boys as well as 14 girls. All HCs conformed to the following standards: (i) an absence of abnormal MRI in the brain; (ii) no ophthalmic surgery history and best-corrected VA not greater than 0 logMAR units; (iii) a state of sanity; (iv) no MRI examination contraindications (like a cardiac pacemaker or implanted metal devices). Our study has gained the approval of the Medical Ethics Committee of the First Affiliated Hospital of Nanchang University, and the protocol adhered to the principles of the Declaration of Helsinki. All participants (including the child and their parents) gave informed consent and details of the objectives of the research, and the latent danger to patients were explained in detail.

### Magnetic Resonance Imaging Parameters

We used a 3-T magnetic resonance scanner (Trio, Siemens, Munich, Germany) to undergo the MRI scanning. During the entire scanning process, we asked all participants to breathe smoothly and remain their eyes closed, but keep awake. A three-dimensional spoiled gradient recalled echo sequence was applied to collect the data. Relevant details about the apparatus are as follows: 176 structural images (gap: 0.5 mm; repetition time (TR): 1,900 ms; echo time (TE): 2.26 ms; thickness: 1.0 mm; field of view: 250 × 250 mm; flip angle: 9°; acquisition matrix: 256 × 256). In addition, 240 functional images (TR: 2,000 ms; TE: 30 ms; thickness: 4.0 mm; gap: 1.2 mm; field of view: 220 × 220 mm; flip angle: 90°; acquisition matrix: 64 × 64; 29 axial) were likewise acquired. The duration time of the whole scanning process is 15 min.

### Functional Magnetic Resonance Imaging Data Processing

Firstly, the MRIcro software^1^ was applied to analyze the collected data. Then, we used the Data Processing Assistant for rs-fMRI software (DPARSF)^2^ and the Statistical Parametric Mapping 8 (SPM8) to preprocess the received information. We removed the data of the first 10 time points to eliminate interference which may be caused by an unsteady magnetic field. Furthermore, slice timing was carried out to correct time differences.

Owing to the differences in brain volume and structure between subjects, spatial standardization was used to process the available images. During this process, we unified the images according to the Montreal Neuroscience Institute standard (MNI152_T1_3mm. nii), and the voxels were immediately re-sampled with a resolution of 3 mm × 3 mm × 3 mm. To dislodge the linear chemotactic effect produced while the subject adapts to the scanning environment, the linear drift was eliminated. Eventually, to reduce high-frequency physiological noise, such as the heartbeat or respiration, only data between 0.01 and 0.08 Hz were collected.

### Statistical Analysis

We used the SPSS 20.0 software (IBM Corporation, Armonk, NY, United States) to compare the ReHo values of certain brain areas in the SA and HC groups, and used the two-sample *t*-test and the Representational state transfer (REST) software to analyze distinctions between this two groups. When the *p*-value < 0.05, it was deemed as statistically significant. The collected data were compared and analyzed by AlphaSim. Corrected thresholds were set at *P* < 0.01, and the cluster size at > 40 voxels. Then, the REST software is used to identify brain regions with significantly changed ReHo values as regions of interest (ROI). The mean ReHo of all voxels in each brain area was taken as the ReHo value of this ROI. In addition, the Pearson correlation analysis was applied to distinguish between the ReHo value and the degree of strabismus in SA individuals.

## Results

### Demographics and Visual Measurements

There are no significant differences were observed in gender (*p* > 0.999) and age (*p* = 0.902) between the two groups. However, significant differences appeared in the best-corrected VA of both eyes (*p* = 0.003 and *p* = 0.004, respectively) (Table 2).

**TABLE 2:** Participant characteristics.

Condition	SA	HCs	*t*	*P*-value*
Male/female	14/10	14/10	N/A	>0.999
Age (years)	8.21 ± 2.24	8.43 ± 1.97	0.256	0.902
Weight (kg)	20.76 ± 2.54	21.17 ± 3.64	0.365	0.891
Handedness	24R	24R	N/A	N/A
Duration of ON (years)	8.21 ± 2.24	N/A	N/A	N/A
BCVA-left eye	0.15 ± 0.05	0.95 ± 0.20	–3.654	0.003
BCVA-right eye	0.20 ± 0.05	1.10 ± 0.15	–3.217	0.004
IOP-L	15.54 ± 4.67	15.47 ± 4.19	0.586	0.932
IOP-R	15.85 ± 5.43	16.11 ± 5.12	0.612	0.901

Independent t-tests comparing the two groups (*p < 0.05, represented statistically significant differences). Data shown as mean standard deviation or n.

*BCVA, best-corrected visual acuity; HCs, healthy controls; IOP, intraocular pressure; L, left; N/A, not applicable; R, right; SA, strabismus amblyopia.*

### Regional Homogeneity Differences

**FIGURE 2:**
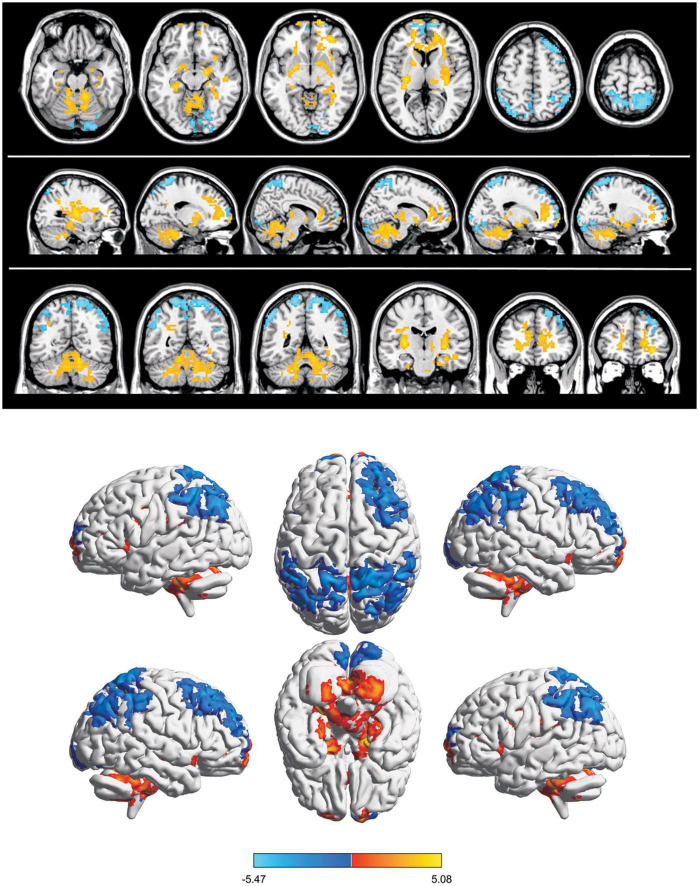
Spontaneous brain activity in SA group. Blue regions (right cerebellum, left frontal superior gyrus, and left putamen nucleus) indicate lower reHo values, whereas red regions (right parietal superior gyrus, left parietal superior gyrus, right lingual gyrus, right frontal superior medial gyrus, and right parietal inferior) show higher ReHo values (AlphaSim-corrected, *P* < 0.05,cluster size > 40).

**FIGURE 3:**
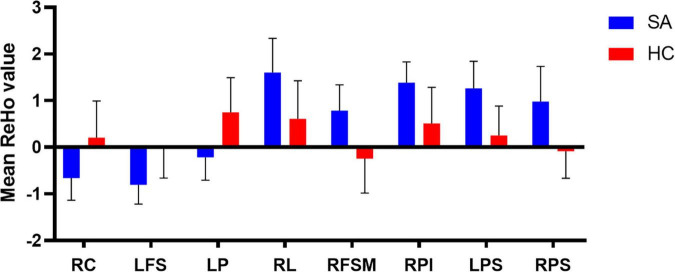
The mean ReHo values in different brain regions in SA and HC groups. RC, right cerebellum; LFS, left frontal superior; LP, left putamen; RL, right lingual; RFSM, right frontal superior medial; RPI, right parietal inferior; LPS, left parietal superior; RPS, right parietal superior.

**FIGURE 4:**
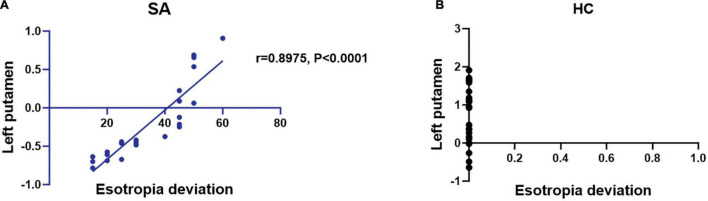
The ReHo value of brain activity in SA group. **(A)** The esotropia deviation is in proportion to ReHo value in left putamen (*r* = 0.8975, *P* < 0.0001). **(B)** The esotropia deviation is disproportionate to left putamen. SA, strabismus amblyopia; HC, healthy control.

Compared with the HC group, the mean ReHo values of the following brain areas in the SA group were remarkably increased: right lingual (RL), right superior frontal medial (RSFM), bilateral superior parietal (SP), and right inferior parietal (RIP) [Figure 2 (red areas), Table 3]. However, the ReHo values of the right cerebellum (RC), left putamen (LP), and left superior frontal (LSF) gyrus were remarkably decreased in the SA group [Figure 2 (blue areas), Table 3]. The comparison of the ReHo values in two groups are presented in Figure 3. Through analysis, there was a positive correlation between esotropia degree and ReHo values of the left putamen (*r* = 0.8975, *p* < 0.0001) (Figure 4).

**TABLE 3:** Brain areas with significantly different ReHo values between two groups.

Brain area	MNI coordinates			BA	Peak voxels*	*T*-value	*P*-values
	
	X	Y	Z				
**SA > HC**							
RLG	21	–75	–12	18	275	–4.16	0.004
RFSMG	12	66	9	8	749	–5.22	< 0.001
RPIG	54	–45	54	40	451	–4.66	0.003
LPSG	–18	–54	69	7	714	–5.47	< 0.001
RPSG	18	–48	72	7	388	–5.05	< 0.001
**SA < HC**							
RC	12	–45	–21		1600	4.5	0.003
LFSG	30	45	–6	13	1548	4.95	< 0.001
LPN	–30	–18	6	13	749	5.08	< 0.001

*The statistical threshold was set at voxel with P < 0.05 for multiple comparisons using false discovery rate.*

*Peak voxels: number of voxels in each cluster.

*SA, strabismus amblyopia; HC, healthy control; MNI, Montreal Neurological Institute; BA, Brodmann’s area; RLG, right lingual gyrus; RFSMG, right frontal superior medial gyrus; RPIG, right parietal inferior gyrus; LPSG, left parietal superior gyrus; RPSG, right parietal superior gyrus; RC, right cerebellum; LFSG, left frontal superior gyrus; LPN, left putamen nucleus.*

## Discussion

**FIGURE 5:**
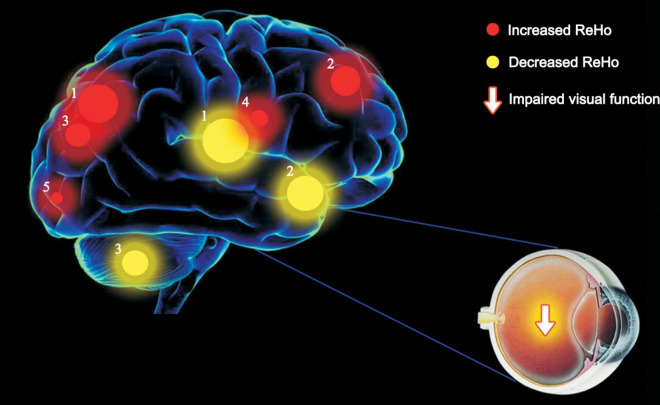
The ReHo values of the altered brain regions. Variable degree of the ReHo values in SA group of the following regions were decreased: 1- Left putamen; (*t* = 5.08), 2- Left frontal sup (BA 13, *t* = 4.95), 3- Right cerebellum (*t* = 4.5). The ReHo values of the following brain regions were higher than HCs: 1- Left parietal sup (BA 7, *t* = –5.47), 2- Right frontal sup medial (BA 8, *t* = –5.22), 3- Right parietal sup (BA 7, *t* = –5.05), 4- Right parietal inf (BA 40, *t* = –4.66), 5- Right lingual (BA 18, *t* = –4.16). The degree of quantitative change was indicated by the size of spots. ReHo, regional homogeneity; HCs, healthy controls; BA, Brodmann’s area.

Children with SA showed increased ReHo values in the RL, RSFM, RIP, and SP areas compared with the HCs, while the mean ReHo values for the RC, LSF, and LP regions were significantly lower (Figure 5).

The lingual gyrus is located in the occipital lobe and has connections with the parahippocampal and the fusiform gyrus. It is a crucial part of the ventral visual stream, which processes visual details, like color, form, and size, processes complex visual stimuli by identifying essential characteristics. Therefore, this area is vital for visual attention and judgment. Earlier research reported an increased ReHo value of the lingual gyrus in patients with concomitant strabismus (CS) (Huang et al., 2016). In our study, an increased ReHo value of the RL was also detected in children with SA, which could be explained by visual compensation.

The frontal lobes are the largest cortical region in the human brain. It is also regarded as a very vital and the most complex area because it has extraordinary rich connections (including afferent and efferent connections) with almost all other parts of the central nervous system (Nauta, 1972). Especially, it is involved smooth pursuit eye movement (Heide et al., 1996). The early abnormal visual conditions experienced by the SA children may disturb the neurodevelopmental processes as well as brain maturation, these changes may lead to a more terrible binocular vision and visual acuity. Therefore, the abnormal changes of ReHo values in frontal lobes may be one reason which causes declined visual function in children with SA.

Parietal lobules are somatosensory areas, which integrate information about feeling, touch, and vision, and facilitate the recognition and recall of size, shape, texture, and the weight of objects. A previous study has confirmed that the parietal lobe has a strong relationship with the visual cortex (Hishida et al., 2019). Ouyang et al. (2017) investigate the parietal lobes in patients with CS using voxel-based morphometry and recognized that compared with the HCs, the volume of the gray matter was reduced in the parietal occipital lobes. The increased ReHo value in this study may be the expression of compensatory brain development.

The cerebellum is involved in motor and balance control, including precise eye movements (Herzfeld et al., 2015). The V1 lobule of the cerebellum is related to spatial vision tasks and has extensive fiber crossing with other areas of the brain. Similarly, the cerebellum is considered as a vital area that controls the movement of the eyes and hands (Nitschke et al., 2005). In another study, a relationship between activation of the cerebellar vermis and visually guided saccades was reported (Hayakawa et al., 2002). A previous research demonstrated that the posterior interposed nucleus located in the cerebellum is an essential brain area in the process of conjugate eye movement in monkeys with strabismus (Joshi and Das, 2013). The observed declined ReHo value of this area may due to visual dysfunction caused by SA, which leads to significant functional brain activity alternations.

The putamen plays an integral role in the learning and memory system, as well as in the processing of visual information (Romero et al., 2008). Lee et al. (2006) used a visuomotor task to detect neuronal activity changes, they found significantly activated signals in the putamen with or without motor-related stimuli. In this study, dropped ReHo value was found in the left putamen in children with SA (Table 4), which could be the result of the visual defect in SA patients.

**TABLE 4:** Brain regions alternation and its potential impact.

Brain regions	Experimental result	Brain function	Anticipated results
Lingual gyrus	SAs > HCs	component of the ventral visual stream, process information	Visual hallucination
Frontal superior medial gyrus	SAs > HCs	Associated with ocular diseases	forced grasping reflex, groping reflex
Parietal inferior gyrus	SAs > HCs	production, expression and reception of language	Body schema disorder, Gerstmann syndrome
Parietal superior gyrus	SAs > HCs	Cortical sensation like stereognosis and two point discrimination	Cortical sensation deprivation
Cerebellum	SAs < HCs	balance and motor control, execution of accurate eye movements	Cerebellar ataxia
Frontal superior gyrus	SAs < HCs	Control autocinesis, language, affection	paralysis
Putamen nucleus	SAs < HCs	Regulate muscle tone, coordination of fine activities	Hyperexplexia, movement disorder

*HCs, healthy controls; SA, strabismus amblyopia.*

In addition, we also found some statistically significant voxels in MRI images in white matter. In previous MRI studies, activated signals are often found in the white matter of the brain. However, whether this signal has the significance for some potential neural activities has been controversial. White matter contains connecting fibers specialized in processing signals between different brain regions, accounting for about half of the brain (Harris and Attwell, 2012). Most of the reports on MRI activation of brain white matter involve the corpus callosum. The corpus callosum contains the largest white matter tract in the brain, which is involved in the transmission of information between the two cerebral hemispheres (Aboitiz et al., 1992), including cognitive, motor, auditory and visual information (Goldstein et al., 2022). Some studies reported that pathways related to visual-motor interhemispheric transfer tasks were observed in the knee of the corpus callosum (Tettamanti et al., 2002; Omura et al., 2004; Weber et al., 2005; Gawryluk et al., 2009). Fabri et al. (2011) found that the posterior part of the corpus callosum can be activated by visual stimulation by performing different tasks on healthy subjects, which is consistent with the previous results of the interhemispheric transfer task (Gawryluk et al., 2011a). In addition, fMRI activation has also been reported in the internal capsule. Studies have shown that activation can be detected in the inner capsule when performing motor tasks (Gawryluk et al., 2011b; Mazerolle et al., 2013). Moreover, the activation signal of fMRI can also be detected in the white matter of the healthy control group and Alzheimer’s disease group during a memory task (Weis et al., 2011).

In this study, we speculate that the activated white matter signal may be a compensatory development of the brain of children with SA to compensate for the abnormal visual experience. In addition, it has been reported that in childhood and early adolescence, the development of the whole brain’s white matter tract will increase, which contributed to improving cognitive ability (Barnea-Goraly et al., 2005). Therefore, the increased white matter signal found in this paper may also be the physiological result of brain development. Among the rapidly growing published MRI articles (Bandettini, 2012), the articles related to the activation of white matter MRI imaging are still relatively rare. We hope to have more research on the white matter function and its abnormalities.

## Conclusion

In summary, the abnormal spontaneous brain activity in children with SA demonstrated in the present study could be attributed to both the development of SA and resultant visual compensation.

From the second trimester of pregnancy, the volume of gray matter brain cells increased rapidly and peaked before the age of six. Similarly, the volume of subcortical gray matter peaked at 14.5 years old. From the second trimester of pregnancy to early childhood, the volume of white matter also increased rapidly (Bethlehem et al., 2022). Therefore, in early childhood, the brain can continuously, quickly and completely compensate for some abnormalities (Benton and Tranel, 2000), resulting in compensatory structural abnormalities of the brain. In addition, if the dominant eye is wounded, or if the other eye is subsequently affected by a disease or disorder, permanent monocular visual impairment observed in amblyopia can become a risk factor for blindness (Harrad and Williams, 2002). Therefore, early treatment of this disease is vital (Fu et al., 2014). The findings of this study lay a foundation for further research into the discovery and diagnosis of SA. Furthermore, this study offers important information to gain a better understanding of SA and provides new insights for treatment.

## Data Availability Statement

The raw data supporting the conclusions of this article will be made available by the authors, without undue reservation.

## Ethics Statement

The studies involving human participants were reviewed and approved by the Medical Ethics of The First Affiliated Hospital of Nanchang University (No: 2020038). Written informed consent to participate in this study was provided by the participants’ legal guardian/next of kin. Written informed consent was obtained from the minor(s)’ legal guardian/next of kin for the publication of any potentially identifiable images or data included in this article.

## Author Contributions

S-WT, G-QC, and Q-YL: conceptualization. S-WT, YG, and Y-CP: methodology. S-WT, L-JZ, and Q-MG: formal analysis and investigation. S-WT, H-YS, X-JZ, and YS: writing—original draft preparation. S-WT, G-QC, and YS: writing—review and editing. YG and YS: funding acquisition. S-WT, H-YS, and YS: resources. S-WT and Y-CP: supervision. All authors contributed to the article and approved the submitted version.

## Conflict of Interest

The authors declare that the research was conducted in the absence of any commercial or financial relationships that could be construed as a potential conflict of interest.

## Publisher’s Note

All claims expressed in this article are solely those of the authors and do not necessarily represent those of their affiliated organizations, or those of the publisher, the editors and the reviewers. Any product that may be evaluated in this article, or claim that may be made by its manufacturer, is not guaranteed or endorsed by the publisher.

## Footnotes

1
http://www.MRIcro.com

2
http://rfMRI.org/
