# Supplementary figures and images for: Altered Brain Activity in Strabismic Amblyopic Children as Determined by Regional Homogeneity: A Resting-State Functional Magnetic Resonance Imaging Study
Source: Front Neurosci. 2022 Jun 2;16:879253. doi: 10.3389/fnins.2022.879253 (PMC9201242; doi:10.3389/fnins.2022.879253)

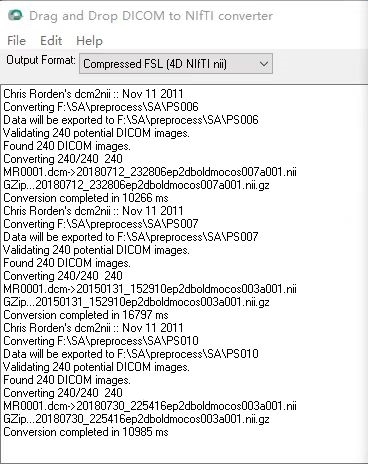

Supplement: Supplementary file 1 [file Image_1.JPEG]

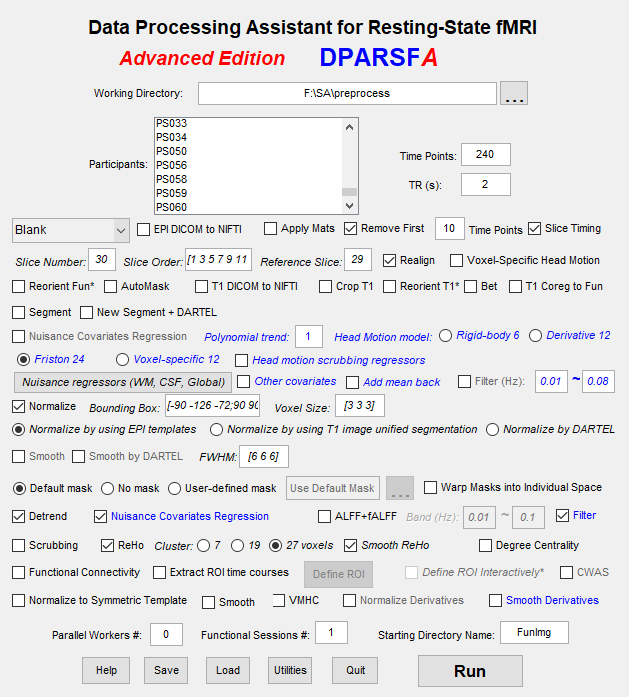

Supplement: Supplementary file 2 [file Image_2.JPEG]

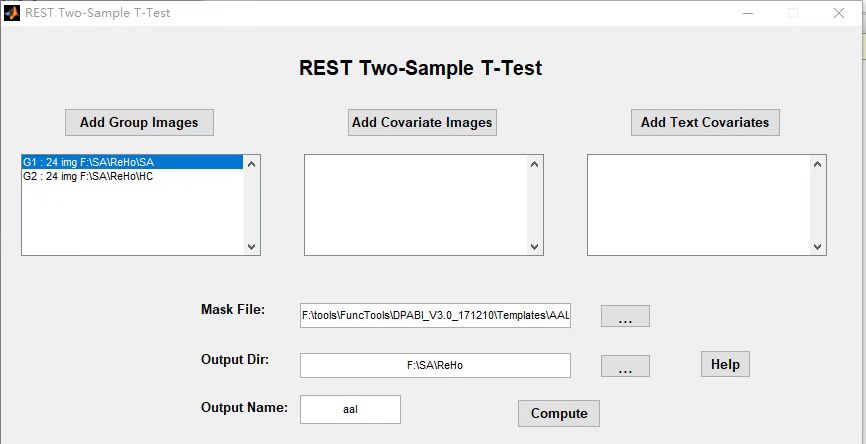

Supplement: Supplementary file 3 [file Image_3.JPEG]

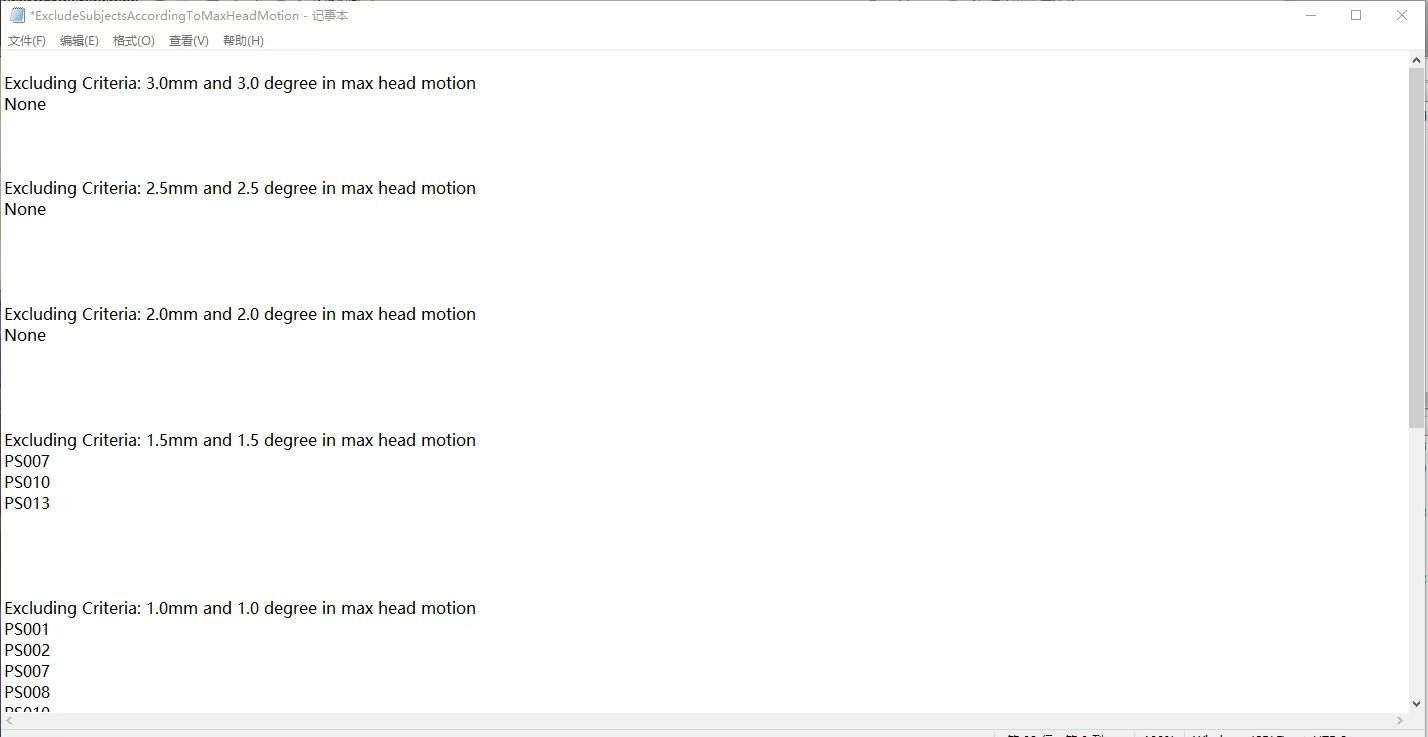

Supplement: Supplementary file 4 [file Image_4.JPEG]

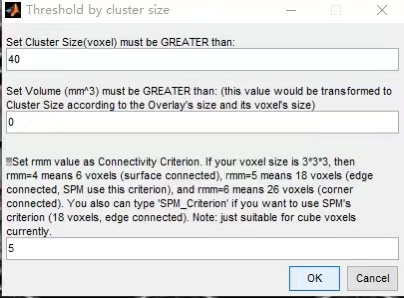

Supplement: Supplementary file 5 [file Image_5.JPEG]

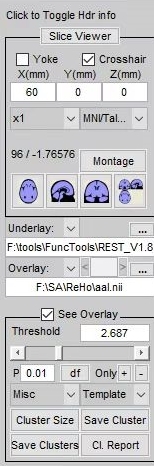

Supplement: Supplementary file 6 [file Image_6.JPEG]
